# Efficacy of traditional Thai massage as adjunctive therapy in patients with major depressive disorder

**DOI:** 10.34172/hpp.42625

**Published:** 2024-07-29

**Authors:** Warangkana Chompoopan, Wichai Eungpinichpong, Suwanna Arunpongpaisal, Worawut Chompoopan

**Affiliations:** ^1^Sirindhorn College of Public Health Khon Kaen, Faculty of Public Health and Allied Health Sciences, Praboromarajchanok Institute, Nonthaburi, Thailand; ^2^Research Center in Back, Neck, Other Joint Pain and Human Performance, Khon Kaen University, Khon Kaen, Thailand; ^3^Faculty of Associated Medical Sciences, Khon Kaen University, Khon Kaen, Thailand; ^4^Faculty of Medicine, Khon Kaen University, Khon Kaen, Thailand

**Keywords:** Massage therapy, Depressive disorder, Complementary therapies

## Abstract

**Background:**

Major depressive disorder (MDD) is the most common mental ailment. Moreover, it is one of the most incapacitating medical conditions. Although antidepressant medication has traditionally been the mainstay of treatment, adjunctive therapy may provide therapeutic advantages that reduce the severity of depression.

**Methods:**

An experiment using randomization and control groups was undertaken. A total of forty-eight individuals diagnosed with severe depressive illness and undergoing antidepressant medication were selected and randomly assigned to either get traditional Thai massage (TTM) treatment, consisting of 90-minute sessions twice a week for eight weeks, or to be part of the control group, which continued with their regular daily activities. The main assessment tools used were the Hamilton Depression Rating Scale (HAM-D), the Clinical Global Impression-Severity (CGI-S), and the Khon Kaen University Depression Inventory 14 (KKU-DI-14). Secondary outcomes, such as blood pressure (BP) and quality of life measured by The EuroQol-5D-5L (EQ-5D-5L), were assessed both before and after the first therapy, as well as at the last session at the 8th week.

**Results:**

The TTM group showed a statistically significant decrease in the HAM-D score within the eighth week of therapy compared to the control group (5.14 points, 95% confidence interval=2.92 to 7.37 points, *P*<0.001).

**Conclusion:**

These findings suggest that combining TTM with antidepressant medication may effectively reduce depression scores and improve quality of life scores.

## Introduction

 Depressive disorders are the most common mental disease, afflicting more than 300 million people worldwide; thus, depression is one of the leading causes of burden worldwide. Depression was ranked 13^th^ in disability-adjusted life years (DALYs) globally in 2019. Depressive disorder will be the largest cause of disease burden worldwide by 2030.^1^ Common signs of depression have both mental and physical symptoms. Mental symptoms include unhappiness, hopelessness, loss of interest as well as the impact on the individual’s functioning; these symptoms have a strong effect on quality of life and health conditions.

 In addition, currently, only 7%-28% of patients affected by depression receive timely and appropriate care, which is remarkably low.^2^ The improvement rate of treatment on young people with depression was 38% under usual conditions.^3^ Major depressive disorder (MDD) with high severity may affect patients who has suffering with illnesses with long durations. Patients with severe depression are at risk of serious outcomes such as self-harm or suicide.^1^

 Currently, many studies have shown that effective treatments are available. These treatments include anti-depressant medication and psychological treatment (promotion and prevention program) to support patients’ mental health conditions. These services include inpatient and outpatient departments that follow standard treatment protocols.^4^ While treatments can be effective for some patients,^5^ relapse rates are high and a considerable group of patients do not respond to treatment at all.^6^

 Adjunctive treatments are interventions that can help patients manage depression. They can help patients reduce signs and symptoms of depression through behavioral principles,^7^ physical exercise,^8^ and massage.^3^ These types of interventions can also benefit patients’ quality of life and be relaxing. In the past, these adjunctive treatments have not been included in guidelines because effectiveness is not known; the understanding of mechanisms of these treatments are also still not largely understood. This might be the reason that patients with depression often seek out alternative or complementary treatments for depression.^9^

 In many countries, the success of treatment rate is low because there is a lack of services for patients with severe mental disorders.^10^ There are also an increasing number of mental health patients that have disorders strongly affecting their quality of life. In Thailand, the services for depression and suicide are available through the mental health system. Patients can access mental health care and receive standard treatment.^11^ Depression is an important cause of loss of healthly productive years, which is documented through DALYs. Depression is a major mental health problem among Thai population. Mental health care interventions to reduce the burden of depression is important. Researchers are actively exploring adjunctive methods with standard treatment. One of the alternative treatments is traditional Thai massage (TTM), which may reduce symptoms of depressive disorder.^12^

 Hand massage is utilized to prevent both physiological and psychological diseases. The mechanisms of action of massage include stimulation of cells at bio-mechanism, neurological psychophysiological levels. Currently, massage has been shown to have positive outcomes including reducing the somatic and mental symptoms of anxiety^13^ and depression.^14^ Massage can be an effective alternative treatment by applying mechanical pressure to tissue. This stimulation improves blood flow, lymph movement, reduces tension in muscles and connective tissue, improves tendon tone, and improves the patients’ quality of life.^15^

 Thus, this study investigated the effects of TTM which is adjunctive treatment, on reducing patients’ depression score, balancing their cardiovascular system, and improving their quality of life.

## Material and Methods

###  Study design and participants

 We conducted an experimental study at two psychiatric departments in Thailand. MDD patients were randomly assigned to either the treatment or control group. Assignment was double-blinded by clinician and therapist. The treatment group received TTM, while the control group experienced regular daily life without TTM provided by the study.

###  Participants

 We recruited participants from the psychiatric clinic of Khon Kaen University Hospital and the Rajanagarindra Psychiatric Hospital, Khon Kaen Province, Thailand. The researchers assured the participants that we would use their information only for research purposes. A licensed psychiatrist diagnosed MDD for patients in our study using the Diagnostic and Statistical Manual of Mental Disorders, Fifth Edition (DSM-5). The participants in this study who were diagnosed with MDD participated while they were on standard medication. The inclusion criteria of the study were participants: (1) were aged 18 years or older, (2) had mild to severe depression, having score 8 or higher using the Hamilton Depression Rating Scale (HAM-D)^16,17^ based on 17 items. This score satisfied DSM-V specifications for MDD. The exclusion criteria were participants: (1) had another psychiatric disorder such as bipolar disorder, schizophrenia, and personality disorder, (2) had a history of heart disease or received medications that had side effects for the heart; (3) had symptoms or injuries for less than 1 year.

###  Randomization

**Figure 1:**
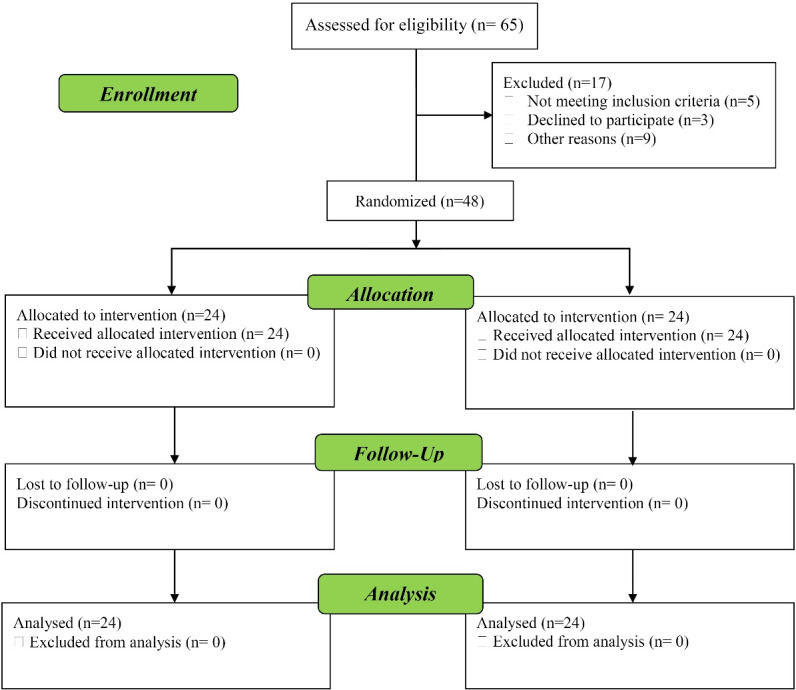
Flow diagram of participants

 A total of 65 patients diagnosed with MDD were randomly assigned to one of the two treatment arms. We used block randomized allocation with block sizes of 4 to attain equal treatment and control group sizes. The flow of participants through our randomized controlled trial (RCT) are shown in Figure 1.

###  Assessment tool

 The Hamilton Rating Scale for Depression (HAM-D) is a tool consisting of 17 items that measures depressive symptoms experienced in the previous week. It has been shown with strong consistency amongst different raters, with an intra-class correlation coefficient (ICC) of 0.92. This demonstrates a strong level of agreement in the scoring process across raters, indicating that the scale successfully measures the fundamental characteristics of depression. Furthermore, the HAM-D demonstrated concurrent validity by exhibiting a strong positive correlation (r = 0.73) with the Beck Depression Inventory (BDI).^18^ Scores are interpreted as follows: 0–7 indicates lack of clinical depression, 8–12 indicates mild depression, 13–17 indicates moderate depression, 18–29 indicates severe depression, and ≥ 30 indicates very severe depression.

 The Khon Kaen University Depression Inventory (KKU-DI) is a self-administered tool for measuring depression severity in Thai adults. It consists of 14 elements and is designed for Thai culture, providing a clear scoring system using a concise 3-point Likert scale (0 = absence, 3 = frequency of occurrence virtually every day). The items have internal consistency with a Cronbach’s alpha value of 0.94 and show considerable stability over time with test-retest reliability of 0.75 to 0.86.^19^ Interpreting Scores: Established cut-off points guide interpretation based on gender and severity: Mild: Male (5-12), Female (6-12), Moderate: 13-14 (both genders), Severe: 15 and above (both genders).

 The Clinical Global Impression-Severity (CGI-S) tool is used to assess the severity of a patient’s mental illness. It is based on clinicians’ experience and uses a 7-point scale to rate symptoms, behavior, and functional ability. The CGI-S has excellent internal consistency, with a Cronbach’s alpha of 0.998 and a kappa statistic of 0.971. It also has significant convergent validity, with a strong correlation with the established Massachusetts General Hospital Cognitive and Physical Functioning Scale (MGH-CPFQ), r = 0.83.^20^ There are seven categories of scores: normal, borderline, mild, moderate, marked, severe, and extreme. Normal scores indicate the absence of symptoms; borderline indicates subtle signs of a condition; mild symptoms cause minimal distress; moderate symptoms hinder social or work activities; marked symptoms significantly disrupt daily life; severe symptoms severely impact behavior and function; and extreme scores require hospitalization.

 The EuroQol-5D-5L has five dimensions, and each dimension is broken down into five levels. It has a high level of test-retest reliability, with intraclass correlation coefficients (ICCs) ranging from 0.65 to 0.91. The EQ-VAS score changes from the start (T1) to the follow-up (T2) showed ICCs ranging from 0.71 to 0.87.^21^

###  Additional assessments 

 Systolic blood pressure (SBP) and diastolic blood pressure (DBP) were recorded by digital blood pressure monitor. The digital blood pressure monitor was recorded in the room where the TTM session occurs. To reduce bias, the researcher recorded three readings to calculate average mean SBP and DBP. The blood pressure was measured when the participant in a seated position. It was measured 5 minutes before and then again 5 minutes after the TTM.

###  Intervention

**Figure 2:**
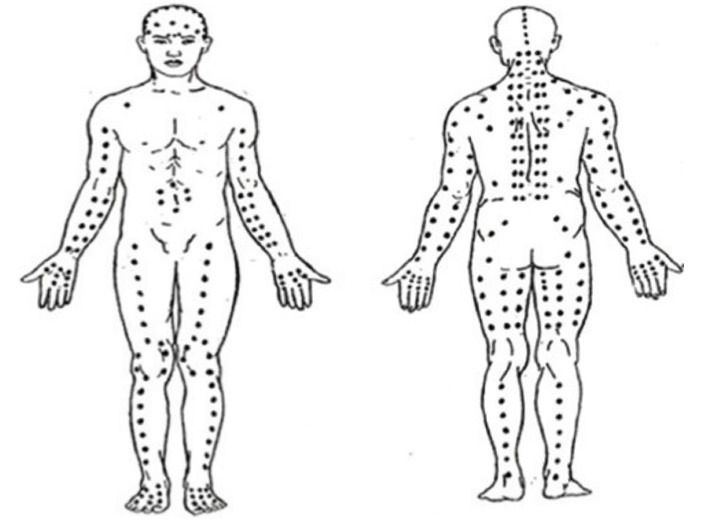
SenSib line of massage

 The technique of TTM used in this study is based on a standard whole-body Thai massage known as SenSib Nuad Thai. The TTM protocol consisted of the following steps: Initially, having the patient lie on his or her back, the massage therapist applied gentle but firm palm pressure to the patient’s medial aspect of the upper arm, aiming to temporally occlude the brachial artery for 20–30 seconds, then released the pressure to let blood flow to the arm and hand. This technique was also done for the lower limb using palm pressure on the femoral artery. This technique is called opening the wind gate, which aims to stimulate blood flow to all the tissues of the limbs. Then the therapist applied gentle but deep thumb pressure massage along the ten meridian lines of TTM that covered major muscles of the limbs, back, neck, and head consequently. The thumb pressure massage along each line was repeated five times. The amount of thumb pressure on each of the body parts was adjusted by the therapist according to, but not exceeding, the pressure pain threshold of the patient. Therapists refrain from utilizing the feet, elbows, or knees to apply pressure to the patients’ body parts. Therapists administered a 90-minute complete body massage in this investigation. The principle of energy lines forms the foundation of this basic total-body TTM. It is known as SenSib lines of massage (Figure 2). At the end of the massage session, the therapist applied a gentle stretch to those muscles, including the calf, hamstring, quadriceps, pectorals, back, neck, shoulder, arm, forearm, and finger muscles. The TTM session covered one and a half hours.

###  Statistical analysis

 Data were analyzed using SPSS for Windows Version 19 (IBM Corp. Released 2010, IBM SPSS Statistics for Windows, and Version 19.0. Armonk, NY: IBM Corp.). Demographic data were summarized using mean ± standard deviation (SD), and percentage. An analysis of covariance (ANCOVA) was performed to take account of chance imbalances at baseline between the groups, using baseline as a covariance variable. This research was conducted to assess the differences in outcome measures between the two groups. Computed the adjusted mean difference and the 95% confidence intervals for each outcome measure in each group and considered p-values below 0.05 as statistically significant.

## Results

###  Demographic characteristics 

 The participant’s demographic information is displayed in Table 1. Women were the majority of the participants. More than half of the participants were single. In both categories, the bulk of participants are between the ages of twenty and thirty. A sizable portion of the participants had completed their bachelor’s degree. 87.50% of participants in both groups obtained employment. There was no statistically significant difference between the two groups (Table 1).

 There was significant difference in HAM-D score between the treatment group and the control group after the intervention was completed. HAM-D score was lower in the treatment group compared to the control group (Mean difference = 5.14; 95% CI: 2.92 to 7.37*; P* < 0.001). We also observed that the KKU-DI-14 score was lower in the treatment group compared to the control group (Mean difference = 4.72; 95% CI = 1.24 to 8.20; *P* = 0.009). Furthermore, the CGIS score was lower in the treatment group compared to the control group (Mean difference = 0.84; 95% CI = 0.39 to 1.30, *P* = 0.001) (Table 2).

Table 3 shows significant differences in mean SBP between the treatment group and the control groupafter 16 sessions. SBP in treatment group was lower than control group (Mean difference = 7.10; 95% CI: 1.75 to 12.45;* P =*0.01). DBP was also lower in the treatment group versus the control group (Mean difference = 7.16; 95% CI = 3.59 to 10.72; *P* < 0.001). However, the quality-of-life score, EQ-5D-5L, was lower in the treatment group compared to the control group; but the difference was not statistically significant (Mean difference = 0.22; 95% CI -0.38 to 0.81; *P* = 0.30). The quality-of-life score measured by EQ-VAS was higher in the treatment group compared to the control group (Mean difference = 11.92, 95% CI = 3.58 to 20.84, *P* = 0.01).

**Table 1:** Demographic characteristics of the participants (n/group = 24)

**Characteristic**	**TTM group**		**Control group**		***P*** **value** ^a^
	**No.**	**%**	**No.**	**%**	
Gender					0.16
Female	21	87.50	17	70.83	
Male	3	12.50	7	29.17	
Age					0.80
20-30	11	45.83	13	54.17	
31-40	5	20.83	5	20.83	
41-50	8	33.34	6	25.00	
Mean ± SD	34.54 ± 11.01		34.83 ± 13.22		
Marital status					0.76
Single	14	58.33	12	50.00	
Married	9	37.50	10	41.67	
Widowed/separated	1	4.17	2	8.34	
Educational level					0.11
Primary school	12	50.00	5	20.83	
High school	4	16.67	6	25.00	
Bachelor’s degree	8	33.33	13	54.17	
Occupational type					1.00
Student	3	12.50	3	12.50	
Employee	21	87.50	21	87.50	

^a^ *P* value was calculated using the chi-square test.

**Table 2:** Comparison of mean outcome measures for depression in the control and TTM groups at 16 sessions

**Outcome measures**	**Control group** **(Mean±SD)**	**TTM group** **(Mean±SD)**	**Mean difference** **(95% CI)**	***P*** ** value**
Hamilton Rating Scale for Depression (HAM-D)	10.72 ± 5.96	5.58 ± 2.07	5.14 (2.92 to 7.37)	< 0.001
New Khon Kaen University Depression Inventory 14 (KKU-DI-14)	13.02 ± 8.46	8.30 ± 5.54	4.72 (1.24 to 8.20)	0.009
Clinical Global Impression-Severity (CGI-S)	3.38 ± 0.72	2.54 ± 0.88	0.84 (0.39 to 1.30)	0.001

**Table 3:** Comparison of the mean of outcome measures for blood pressure and quality of life in TTM and control groups

**Outcome measures**	**Control group** **(Mean±SD)**	**TTM group** **(Mean±SD)**	**Mean difference** **(95% CI)**	***P*** ** value**
Systolic blood pressure	105.02 ± 8.26	97.92 ± 11.03	7.10 (1.75 to 12.45)	0.01
Diastolic blood pressure	70.95 ± 5.80	63.79 ± 6.36	7.16 (3.59 to 10.72)	< 0.001
EuroQol-5D-5L (EQ-5D-5L)	5.76 ± 1.01	5.54 ± 1.01	0.22 (-0.38 to 0.81)	0.30
EQ-VAS	68.98 ± 17.32	80.90 ± 10.62	11.92 (3.58 to 20.84)	0.01

## Discussion

 Studies have demonstrated that TTM effectively alleviates symptoms of depression. The present study aligns with Arnold’s research on the efficacy of affect-regulating massage therapy combined with progressive muscle relaxation. The findings indicate a reduction in the HAM-D, a widely used psychiatric assessment tool, as well as a visual analog scale.^22^ Nevertheless, variations existed in the study’s timeframe, which spanned 8 weeks, and the frequency of massage treatment sessions, occurring twice per week, with the intention of attaining specified objectives. The treatment group exhibited considerably superior progress in comparison to the control group.

 This study supports the potential benefits of massage for patients with depression. Whether the effects of massage are generalizable to other types of depression, as well as to other individuals with depression, remains to be explored. Massage is a multifaceted treatment that has several intricate physiological and psychological consequences, including the relaxing of both muscles and the psyche. Despite an incomplete comprehension of massage’s methods of action, massage treatment is thought to activate substantial nerve fibers, resulting in alterations in the release of endorphins, oxytocin, and other hormones. Research suggests that massage improves the functioning of the parasympathetic nervous system. Increasing research suggests that massage possesses advantageous mechanisms^23^ that reduce both pain and depression.^24^ Massage therapy might potentially alleviate symptoms of depression in individuals with specific circumstances, such as pregnancy.^25^

 TTM refers to a form of therapeutic intervention that involves the application of deep pressure. The therapist applies a customized amount of force to the patient, determining the specific depth at which the tissue needs pressure to produce optimal relaxation. Second. Those experiencing considerable psychological stress can benefit from massage therapy. It can successfully lower salivary alpha-amylase levels in healthy individuals,^26^ alleviate stress,^27^ and efficiently alleviate muscular tension^28^ and chronic pain.^29^This study investigates the effects of receiving 16 sessions of Thai traditional massage, each lasting 90 minutes, on an individual’s psychological, physiological, and social well-being. Our findings indicate that using TTM alongside antidepressant medication is beneficial for those diagnosed with severe depressive disorder. Our findings indicate that individuals who received Thai traditional massage experienced enhanced, favorable results and a more pronounced impact as compared to the control group.

 Despite the availability of effective treatments, many people with depression struggle to access timely and adequate care. This is concerning, especially considering the increasing use of complementary and alternative therapies like massage for depression, which may not be as effective. This highlights the need for prevention strategies to lessen the overall burden of depression on individuals, families, and society.

 Given the significant impact of depression on health, preventative programs could be a valuable tool for individuals experiencing mild symptoms. Doctors and policymakers should seriously consider this approach.

 Further research is needed to understand the specific needs of different populations, particularly women who experience higher rates of depression compared to men. This will help ensure that preventative programs are effective and accessible to all.

## Limitations

 This study has three key limitations:

1. Limited generalizability: The majority of participants were female, limiting the applicability of the findings to the male population. Further research with a more balanced gender representation is needed to draw broader conclusions.
2. Potential bias: Outcome assessments were not blinded to researchers, introducing potential for bias in data interpretation. Future studies should consider a single-blinded RCT design to mitigate this risk.
3. Confounding effects of medication: Most participants were already taking antidepressants, making it difficult to isolate the specific effects of TTM alone. The study design could not ethically stop ongoing medication, and any changes in medication during the study could have influenced the massage’s impact. Future research could consider exploring massage alongside medication tapering or in medication-free populations, if ethically and medically feasible.

## Conclusion

 This study suggests that TTM may be a beneficial addition to standard antidepressant treatment for depression. Compared to the control group, participants receiving TTM alongside medication experienced significantly lower depression scores and improved quality of life, without any reported adverse effects. However, further research is needed to confirm these findings and explore the long-term benefits of TTM for both depression and other health conditions.

## Acknowledgements

 We also valued good cooperation from the study volunteers. This study was grateful for the help of Khon Kaen University Hospital (Srinagarind Hospital) and Khon Kaen Rajanagarindra Psychiatric Hospital, Khon Kaen province, Thailand for allowing access to the setting and equipment.

## Competing Interests

 The authors assert that there is no conflict of interest.

## Ethical Approval

 The present study was carried out in accordance with the principles outlined in the Declaration of Helsinki. All protocols involving human subjects or patients were granted approval by the Bioethics Committee of the Khon Kaen University Ethics Committee for Human Research (reference number: HE 601073). All subjects provided written permission.
